# Telemedicine Uptake in a Geriatric Assessment Center During the COVID Crisis

**DOI:** 10.1093/geroni/igab046.2405

**Published:** 2021-12-17

**Authors:** Noelle Frye, Margaret Doyle, Richard Marottoli

**Affiliations:** 1 Yale New Haven Health System, Yale New Haven Health System, Connecticut, United States; 2 Yale University, Yale University, Connecticut, United States; 3 Yale University, New Haven, Connecticut, United States

## Abstract

The Yale New Haven Hospital Adler Geriatric Assessment Center is an outpatient consultative service that provides comprehensive assessment of older adults. As elsewhere, at Adler the COVID crisis necessitated a rapid shift in mode of care following a total cessation of in-person visits from late March 2020 to the end of May 2020. While our patients initially preferred telephone visits, video visits as a proportion of total scheduled increased from an average of 6% in the last full week of March to 24% in the last week in May possibly indicating increasing familiarity and comfort with the technology during that time. In addition, while video appointments as a proportion of total scheduled dropped rapidly in June 2020 as face-to-face appointments were reintroduced, we found a steady increase in the proportion of video visits from 3% in the first week of July 2020 to 7% in the second week of February 2021. To test for significance, we ran logistic regression models modelling the dichotomous video-appointment variable as the outcome and week and day of week as continuous variables. We found there was a significant increase in the proportion of appointments delivered over video both during the time when no face-to-face video appointments were allowed (OR=1.21, CI=1.13,1.30) and later in the pandemic (OR=1.04, CI=1.02,1.06). Durbin-Watson statistics were run to ensure that autocorrelation could be ignored. Sensitivity analyses limiting the sample to those with non-cancelled appointments gave similar results. Future analyses will examine patient clinical and demographic characteristics that might influence these trends.

